# Loss of Tctn3 causes neuronal apoptosis and neural tube defects in mice

**DOI:** 10.1038/s41419-018-0563-4

**Published:** 2018-05-03

**Authors:** Bin Wang, Yingying Zhang, Hongli Dong, Siyi Gong, Bin Wei, Man Luo, Hongyan Wang, Xiaohui Wu, Wei Liu, Xingshun Xu, Yufang Zheng, Miao Sun

**Affiliations:** 1 Institute for Fetology, the First Affiliated Hospital of Soochow University, Suzhou City, 215006 Jiangsu China; 2 Institute of Neuroscience, Soochow University, Suzhou City, 215123 Jiangsu China; 3 Department of Neurology, Suzhou Hospital of Traditional Chinese Medicine, Suzhou City, 215123 Jiangsu China; 4 Obstetrics and Gynecology Hospital Research Center, Institute of Reproduction and Development, Fudan University, Shanghai, 200433 China; 5 State Key Laboratory of Genetic Engineering, MOE Key Laboratory of Contemporary Anthropology, and Collaborative Innovation Center for Genetics & Development, School of Life Sciences, Fudan University, Shanghai, 200438 China; 6 Institute of Developmental Biology & Molecular Medicine, Fudan University, Shanghai, 200433 China; 7 Department of Pathology, the First Affiliated Hospital of Soochow University, Suzhou City, 215006 Jiangsu China

## Abstract

Tctn3 belongs to the Tectonic (Tctn) family and is a single-pass membrane protein localized at the transition zone of primary cilia as an important component of ciliopathy-related protein complexes. Previous studies showed that mutations in Tctn1 and Tctn2, two members of the tectonic family, have been reported to disrupt neural tube development in humans and mice, but the functions of Tctn3 in brain development remain elusive. In this study, Tctn3 knockout (KO) mice were generated by utilizing the piggyBac (PB) transposon system. We found that Tctn3 KO mice exhibited abnormal global development, including prenatal lethality, microphthalmia, polysyndactyly, and abnormal head, sternum, and neural tube, whereas Tctn3 heterozygous KO mice did not show abnormal development or behaviors. Further, we found that the mRNA levels of Gli1 and Ptch1, downstream signaling components of the Shh pathway, were significantly reduced. Likewise, neural tube patterning-related proteins, such as Shh, Foxa2, and Nkx2.2, were altered in their distribution. Interestingly, Tctn3 KO led to significant changes in apoptosis-related proteins, including Bcl-2, Bax, and cleaved PARP1, resulting in reduced numbers of neuronal cells in embryonic brains. Tctn3 KO inhibited the PI3K/Akt signaling pathway but not the mTOR-dependent pathway. The small molecule SC79, a specific Akt activator, blocked apoptotic cell death in primary mouse embryonic fibroblasts from Tctn3 KO mice. Finally, NPHP1, a protein with anti-apoptotic ability, was found to form a complex with Tctn3, and its levels were decreased in Tctn3 KO mice. In conclusion, our results show that Tctn3 KO disrupts the Shh signaling pathway and neural tube patterning, resulting in abnormal embryonic development, cellular apoptosis, and prenatal death in mice.

## Introduction

Loss of a single protein component within a ciliary protein complex disrupts the integrity of that complex and causes defects in the membrane protein composition of the cilia^1,2^ that is associated with a group of diseases termed ciliopathies^3^, including Joubert syndrome (JBTS), oral-facial-digital syndrome (OFDS), Meckel-Gruber syndrome (MKS), nephronophthisis (NPHP), and Bardet-Biedl syndrome (BBS). Among causative genes for these diseases, tectonic mutations have been shown to be important in facilitating ciliopathies^4–7^. Tectonic (Tctn) family proteins are a group of proteins existing in the cilium transition zone (TZ), including Tctn1, Tctn2, and Tctn3^8,9^. Tctn proteins are considered to play a vital role in trafficking proteins into the cilia and are required for the development of cilia and ciliogenesis^10,11^, although how Tctn proteins facilitate selective transportation of ciliary proteins to cilia remains unknown. Previous studies have shown that Tctn1 and Tctn2 mutations result in abnormal development in mice, such as defects in eye development, neural tube, and limbs^8,12^. Owing to the high sequence homology of the three Tctn members, Tctn3 mutations may share similar phenotypes if the functions of Tctn3 are not redundant with those of Tctn1 and Tctn2. So far, the functions of Tctn3 remain unclear, requiring further investigation.

Previous studies demonstrated that Tctn1, Tctn2, and Tcnt3 act as regulators of the sonic hedgehog signaling (Shh) pathway^6,8,12^, which is essential for cell fate and organ development^13–17^. Specifically, both in tectonics mutated primary mouse embryonic fibroblasts (pMEFs) and mice, Ptch and Gli1, widely used transcriptional targets of the SHH pathway, decline significantly^8,9,12^. Studies have shown that Tctn1 is epistatic to Ptch, Rab23, and Smo^8^; however, the exact role of Tctn3 in the SHH signaling pathway remains elusive. To date, increasing evidence shows that Shh determines cell fate by regulating the PI3K/Akt and mTOR signaling pathways^18,19^. Nephrocystin-1 (NPHP1) is an adaptor molecule distributed in the cytoplasm and localized to cilia, cell-cell junctions and cell-matrix adhesion sites^20,21^. Both NPHP1 and Tctn3 have been demonstrated to interact with Tctn1 and/or Tctn2^4,10,12^. Interestingly, NPHP1 regulates resistance to apoptosis^22^; however, the elaborate mechanism of this regulation remains largely unknown. Given that both Tctn3 and NPHP1 localize at the TZ of the cilia and some of their interactors are overlapping^23^, we hypothesized that Tctn3 mutant mice would have abnormal development and increased apoptosis by affecting Shh and PI3K/Akt or mTOR signaling pathways. We further hypothesized that increasing levels of apoptosis would probably be associated with expression of NPHP1.

In this study, to examine the function of the Tctn3 protein in CNS development and its effect on cell fate, we generated a Tctn3 KO mouse model using the piggyBac (PB) transposon system. We found that Tctn3 KO results in increased apoptosis, abnormal brain development, and prenatal death in mice by altering Shh and PI3K/Akt signaling pathways.

## Materials and methods

### Animals

Tctn3 mutant mice were generated by insertional mutagenesis in the C57 background. Mice carrying single-copy PB transposon PB [Act-RFP] in the genome were mated with mice carrying transposase Act-PBase^24^. The mice were produced by conventional pronuclei injection of linear plasmids to ensure co-integration of both donor and helper plasmids in the same site. Double positive male offspring were mated with wild-type mice to generate transposase-negative mutants that carry remobilized PB insertions. The Tctn3 mutant line was one of the mutagenesis lines generated in the offspring. The mutation was mapped by inverse PCR as described^25^. Experimental mice were raised in specific pathogen-free animal housing at the experimental animal center of Soochow University. All mice were placed in cages with a maximum of five mice and 12 h of light (7 am to 7 pm) /dark (7 pm to 7 am) cycle. Behavioral tests were performed at the light cycle with illumination background of 120 lux. All procedures and protocols in this study were approved by the Institutional Animal Care and Use Committee of Soochow University and followed the Guidelines for the Care and Use of the National Institutes of Health (NIH).

### Western blot analysis

Mouse brains at embryonic day 12.5 (E12.5) were collected and homogenized utilizing lysis buffer containing protease inhibitor cocktails. After homogenates were centrifuged at 4 °C, supernatants were collected, and protein concentrations were determined. Samples (30–50 μg) in sample buffer were separated on sodium dodecyl sulfate-polyacrylamide gel electrophoresis. Subsequently, proteins were transferred to membranes (Millipore, Germany), and membranes were blocked with 5% milk in phosphate-buffered saline/0.1% Tween20 (PBST) for 1 h to remove non-specific binding. Membrane blots were incubated with primary antibodies overnight with shaking at 4 °C. After washing, membrane blots were incubated with horseradish peroxidase-conjugated secondary antibodies at room temperature for 1 h. The method of detection of immunoreactive proteins was performed as previously described^26^. Protein signals were visualized using the ECL chemiluminescence system (Thermo Company, West Chester, Pennsylvania, USA) and detected on medical X-ray films. Films were scanned, and densitometric analysis of the bands was performed with AlphaEase Image Analysis Software (Version 3.1.2, Alpha Innotech). Primary antibodies were rabbit polyclonal NeuN (Abcam, ab177487,1:1000, Cambridge, MA, USA); rabbit polyclonal anti-phospho-Akt (Ser473) (4060S, 1:1000), anti-mTOR (2983S, 1:1000) and anti-phospho-mTOR (Ser2448) (5536S, 1:1000) (Cell Signaling Technology Inc., Danvers, MA, USA); rabbit polyclonal anti-Akt (YT0178, 1:1000); rabbit polyclonal anti-NPHP1 (YN0942, 1:1000) and rabbit polyclonal anti-(ADP-ribose) polymerase-1 (PARP-1, YC0073,1:1000, ImmunoWay Biotechnology Company, Plano, TX, USA); rabbit polyclonal anti-Bax (BS6420, 1:500, Bioworld Technology Inc., St Louis Park, MN, USA); rabbit monoclonal anti-Bcl-2 (A5010, 1:1000, Selleckchem, Houston, TX, USA); mouse monoclonal anti-tubulin (T8328 1:20000) (Sigma, St. Louis, MO, USA); and mouse monoclonal anti-GAPDH (sc-32233, 1:1000, Santa Cruz Biotechnology, Delaware, CA, USA).

### Quantitative PCR analysis (Q-PCR)

Total RNA was extracted from mouse brains at E12.5 with an RNeasy Plus Mini kit according to the instructions. Reverse transcription was performed using a Transcriptor First Strand cDNA synthesis Kit (Roche, Germany) as described previously^27^. Real-time PCR was performed in a 20 μl volume using a 7500 real-time PCR machine (Applied Biosystems, USA). For a 20 μl reaction, 50 ng of single-stranded cDNA was used. The following primers were used: Gli1 forward primer, 5′-CCAAGCCAACTTTATGTCAGGG-3′, Gli1 reverse primer, 5′-AGCCCGCTTCTTTGTTAATTTGA-3′; Ptch1 forward primer, 5′-AAAGAACTGCGGCAAGTTTTTG-3′, Ptch1 reverse primer, 5′-CTTCTCCTATCTTCTGACGGGT-3′; Tctn3 forward primer, 5′-ATCGCAGTCCTTGTCACAGCAG-3′, Tctn3 reverse primer, 5′-CAGCTCCTCCGGATTCGTCTG-3′; GAPDH forward primer, 5′-CATGGCCTTCCGTGTTCCTA-3′, GAPDH reverse primer, 5′- CTTCACCACCTTCTTGATGTCATC-3′. GAPDH was used as an internal control. The relative ratio of mRNA was calculated using the 2^−ΔΔCt^ method. Each sample was run in triplicate, and the average value was calculated.

### Immunofluorescent staining

For immunofluorescent staining, E10.5 embryonic brains were fixed in 4% paraformaldehyde. After dehydration in 30% sucrose, the embryonic brains were cut into sections with a frozen cryostat at −20 °C. Frozen brain slices were mounted on glass. After blocking with PBS containing 0.1% Triton X-100 and 1% heat-inactivated goat or sheep serum for 10 min, the slices were incubated with primary antibodies at 4 °C overnight. On the second day, sections were washed with PBS and then incubated with secondary antibodies at room temperature for 1 h. Images were digitized under a fluorescence microscope (Axio ScopeA1, Carl Zeiss, Oberkochen, Germany) with a camera. The primary antibodies used in this study as were as follows: Olig2 (Millipore, Billerica, MA, USA 1:200); Shh, Foxa2, Nkx2.2, Hb9, Isl1/2, Pax6, and Pax3 (Developmental Studies Hybridoma Bank, IA, USA 1:10); and Arl13b (Proteintech, IL, USA 1:500).

### Immunoprecipitation

Fresh brain tissues were homogenized in lysis buffer, and homogenates were centrifuged at 16,000 × *g* for 30 min. The protein concentration of supernatants was determined. Samples containing 500 μg protein in 500 μl were pre-absorbed with protein A agarose beads (Santa Cruz Biotechnology, Delaware, CA, USA) for 1 h at 4 °C with gentle shaking. After centrifuging, the supernatants were incubated with 5 μl rabbit polyclonal anti-Tctn3 (Proteintech, IL, USA) antibody or control rabbit IgG at 4 °C overnight. On the second day, Protein A beads (50 μl) were added and incubated for 2 h at room temperature. Further, samples were centrifuged for 2 min at 1000 × *g* at 4 °C. Beads were further washed with PBS three times. The bound proteins were eluted with sample buffer containing 100 mM Tis-HCl (pH 6.8), 4% sodium dodecyl sulfate, 0.2% bromochlorophenol blue, 200 mM dithiothreitol, and 20% glycerol and were heated at 96 °C for 10 min. Eluates were collected for Western blot analysis.

### Cell culture

Tctn3 Het and KO mouse pMEFs were prepared from E12.5 mouse embryos. The pMEFs were cultured in DMEM supplemented with 10% FBS (fetal bovine serum), 1% penicillin-streptomycin at 37 °C and 5% CO_2_. When cells reached 50–60% confluence, they were incubated with DMSO/SC79 (4 μg/ml) for 24 h for apoptosis detection^28,29^.

### Determination of apoptosis by flow cytometry

The pMEFs were incubated with DMSO/SC79 (4 μg/ml) for 24 h. Apoptotic cells were identified using the Annexin V-FITC apoptosis kit (C1063, Beyotime, Nantong, China)^30^. After cells were stained with Annexin V-FITC/PI, flow cytometric analysis was performed using a Becton Dickinson FACS Calibur flow cytometer (BD Biosciences, San Diego, California, USA). Data were analyzed with Cell Quest Pro software (BD Bioscience).

### Skeletal staining

Skeletal staining with alizarin red S and alcian blue was performed as described before^31^. After removing skin and viscera, newborn mice were fixed in 95% ethanol for 5–7 days. Mice were put into alcian blue solution (15 mg alcian blue in 80 ml 95% ethanol and 20 ml glacial acetic acid) and left for 1–2 days. Next, mice were washed with 95% ethanol for another 1 day and washed with a freshly prepared 1% KOH solution until mice appeared quite clear. Embryos were placed in alizarin red S/KOH solution (5 mg of alizarin red S in 100 ml of 2% KOH) until bone was stained purple. The bones were rinsed in a series of mixtures of 2% KOH and glycerol solution (2% KOH: Glycerol = 80:20; 60:40; 40:60; 20:80) and swirled occasionally for several weeks. Specimens were stored in 100% glycerol.

### Elevated plus maze test

The elevated plus-maze was approximately 70 cm above the ground. The maze consists of two opposite open arms (30 cm × 5 cm) and two opposite closed arms that have 14-cm-high opaque walls. At the beginning of the experiment, animals were placed at the center of the plus-maze facing open arms, and their behaviors were recorded for 10 min. After each test, the maze was cleaned with 50% ethanol solution to eliminate mouse odor. The time spent in closed arms and open arms was recorded as previously described^32,33^.

### Open field test

The open field device (40 × 40 × 40 cm^3^) consists of white plastic opaque walls. The mice were placed in the center of the field. Their movements in the field were recorded over a 6-min session. Central entries and the total time spent in the central area (20 × 20 cm^2^) were recorded as previously described^33^.

### Forced swim test

The forced swim test was performed as previously described^34,35^. Each mouse was placed in a 15 cm diameter, 20 cm height glass cylindrical container filled with water (23–25 °C) to a 14 cm depth. Immobility time was calculated as the period during which they stopped struggling. Time spent floating on the water and smallest movement to keep floating on water were also counted. The test was recorded for 6 min.

### Tail suspension test

The tail suspension test was carried out according to previous reports^35,36^ with slight modifications. Mouse tails were taped to the edge of a shelf and suspended 35 cm above the desktop. The immobility time, which excludes the time spent struggling, was recorded using a stopwatch for 6 min.

### Rotarod test

The rotarod apparatus (UGO BASLLE S.R.L, Italy) can reflect coordination performance. The mice were trained for three 5-min trials at 5 rpm on two subsequent days, and the test was carried out on the third day. During the test, the rotating rod was set to speed up from 0 to 40 rpm over 5 min. The latency to fall from the rotarod was averaged over three trials^26^.

### Statistical analysis

All data are expressed as mean ± SEM. GraphPad Prism 5 (GraphPad Software Inc., La Jolla, CA, USA) was used for statistical analysis. Student’s *t* test was used to determine the differences between two groups (factor: genotype). The differences among groups were determined with two-way ANOVA followed by Bonferroni post hoc tests (factors: mouse genotype and treatment). *P* values < 0.05 were considered statistically significant.

## Results

### **Tctn3 KO resulted in abnormal development and prenatal death in mice, with no abnormalities found in Tctn3 heterozygote mice**

**Fig. 1:**
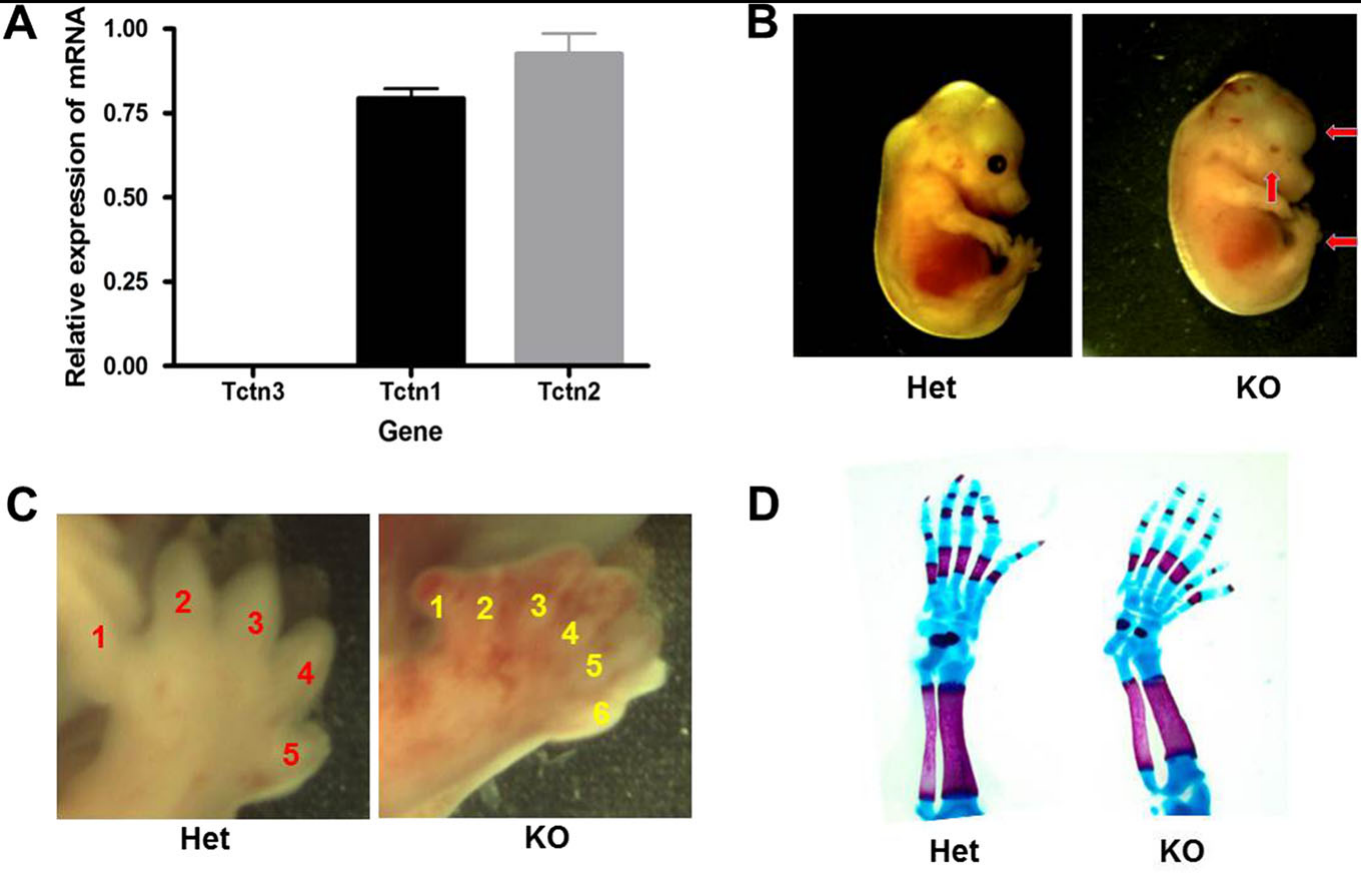
Tctn3 KO resulted in abnormal development and prenatal death in mice. Mouse embryos at E10.5 were collected and total RNA was extracted. The relative mRNA expression of Tctn1, Tctn2, and Tctn3 in Tctn3 Het KO mice and Tctn3 KO mice was detected by quantitative PCR. *N* = 3 (**a**). Mouse embryos at E14.5 were collected, and pictures were taken to examine the head and eyes (**b**). Polysyndactyly phenomenon at E14.5 days was shown (**c**). At postnatal stage P0, mice were collected. Alizarin red S and alcian blue staining was performed. Polysyndactyly was shown in Tctn3 KO mice (**d**)

The role of Tctn3 in the development of mouse embryos is unclear. Tctn3 knockout (KO) mice were generated as described above. Heterozygous Tctn3 mice were crossed to produce Tctn3 KO mice. In all surviving neonates, only 2 Tctn3 KO mice were found but were dead and subsequently used for bone staining. This suggested that Tctn3 KO causes prenatal death. To further explore this, we collected whole embryos at E10.5 and extracted total RNA. DNA samples were also extracted to verify the genotypes. Our results showed that Tctn3 mRNA was not detected in Tctn3 KO mice; however, relative levels of Tctn1 and Tctn2 mRNA were almost unchanged in Tctn3 KO mice (Fig. 1a). These results showed the existence of Tctn3 KO embryos. Therefore, we further examined the phenotypes of Tctn3 KO embryos at E14.5. Nearly half of Tctn3 KO mice were dead at E14.5, and the others died before the birth. The living Tctn3 KO mice were similar in size to Tctn3 heterozygous (Het) KO mice and wild-type (WT) mice. However, abnormal head was observed in Tctn3 KO mice. Parentally, Tctn3 KO mice had small eyes that were kept closed (Fig. 1b). In addition, polysyndactyly was found in Tctn3 KO mice (Fig. 1c,d) as reported in Tctn1/2 KO mice^4,12^. Furthermore, Tctn3 KO mice exhibited dome-shaped heads with a hole, decreased anteroposterior length (Fig. S1A) and abnormal bony fusion of the sternum (Fig. S1B). Since homozygous Tctn3 KO mice died prenatally, we further examined whether there was an abnormal phenotype in the Tctn3 Het KO mice. First, we found that the Tctn3 mRNA level in Tctn3 Het KO mice was unchanged compared to that of wild-type mice (*P* > 0.05, Fig. S2A). Tctn3 Het KO mice did not show embryonic lethality or abnormal development as in Tctn3 KO mice. There was no difference in body weight between Tctn3 Het mice and wild-type (WT) mice at 2 months (*P* > 0.05, Fig. S2B). We further performed a series of behavioral tests to characterize the phenotype of Tctn3 Het KO mice. In the rotarod test, there was no significant difference in the latency to fall between the Tctn3 Het KO mice and WT mice (*P* > 0.05, Fig. S3A). In the forced swim test and the tail suspension test, there was no significant difference in immobility time between Tctn3 Het KO mice and WT mice (*P* > 0.05, Fig. S3B, C). Similarly, no differences in the elevated plus maze test and open field test, two tests for anxious behaviors, between Tctn3 Het mice and WT mice were detected (*P* > 0.05, Fig. S3D-F). These results suggest that Tctn3 KO leads to abnormal development and embryo lethality in mice, but there were no abnormal behaviors or brain abnormalities observed in Tctn3 Het KO mice.

### **Tctn3 KO cause abnormal neural tube patterning in mice**

**Fig. 2:**
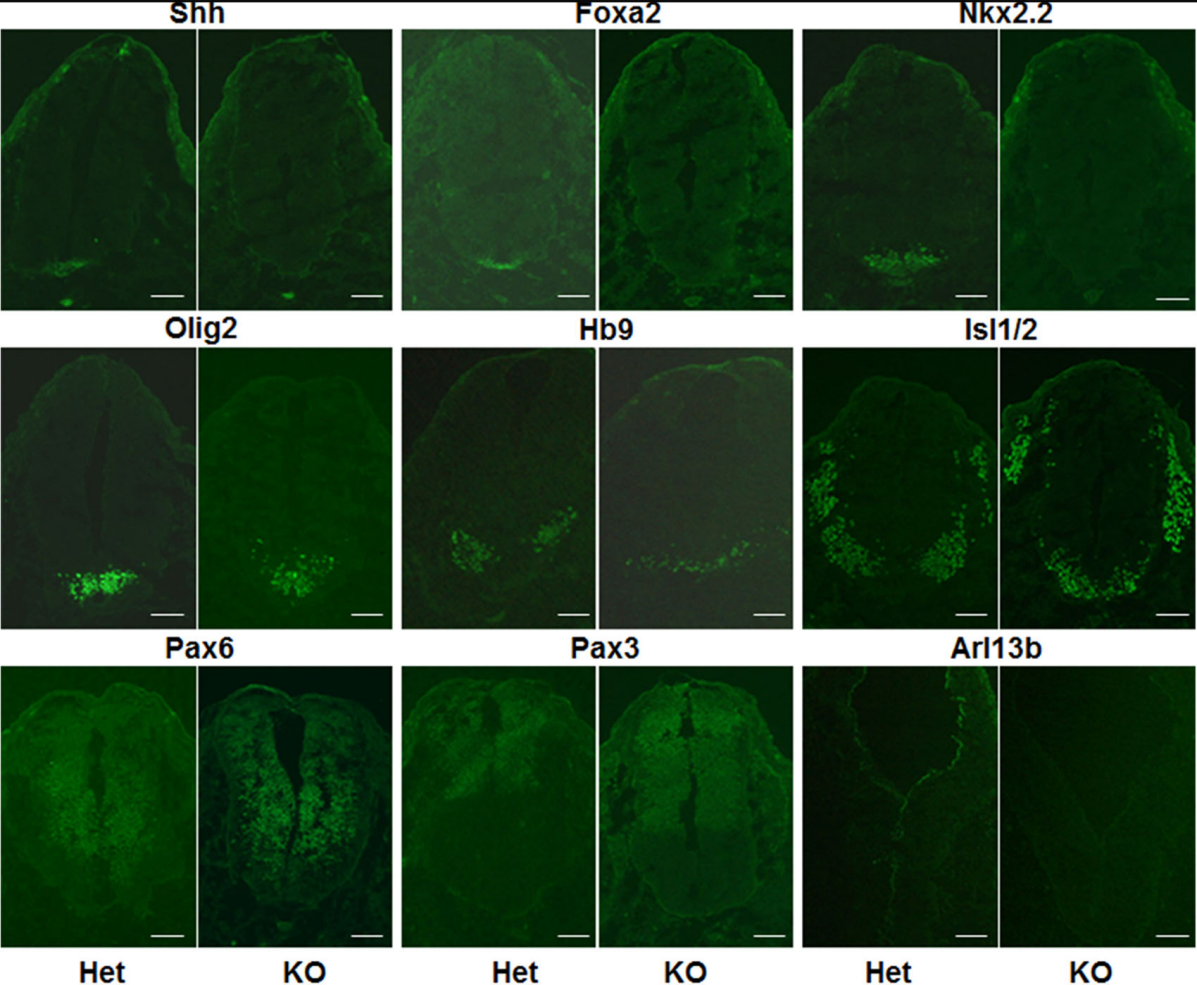
Tctn3 KO caused abnormal neural tube patterning in embryos. Embryonic brains at E10.5 from Tctn3 Het KO mice and Tctn3 KO mice were fixed and cut into sections for staining. Neural tube patterning was examined by fluorescent staining for neuronal markers as indicated. The expression and distribution of Shh, Foxa2, Nkx2.2, Olig2, Hb9 and Isl1/2, Pax6, Pax3, and Arl13b was detected. Scale bars: 50 μm

Tectonic proteins consist of Tctn1, Tctn2, and Tctn3^8^. Tctn1 and Tctn2 were found to play an important role in neural tube patterning by mediating the hedgehog pathway^8,12^. To determine if Tctn3 regulates neural tube patterning as well, we examined neural tube patterning-related proteins in Tctn3 KO embryos. The expression of Shh in the ventral neural tube was significantly decreased in Tctn3 KO embryos. Consistent with Shh expression in Tctn3 mutant mice, Foxa2, which is normally induced by a high level of Shh was also lost in the Tctn3 KO embryos. The expression domain of Nkx2.2, which was positive V3 progenitors specified, shifted ventrally. Olig2, known for determining motor neuron and oligodendrocyte differentiation, shifted ventrally. The patterning and expression of Hb9 or Isl1/2, the motor neuron marker, was affected in Tctn3 KO mice as well. Pax6 expression was also altered, slightly shifting to the ventral side. However, the expression of Pax3 in the dorsal neural tube, which is ventrally limited by low Shh signaling, was unchanged. In addition, Arl13b, a cilia-specific marker, was significantly reduced (Fig. 2).

### Tctn3 KO resulted in abnormal expression of Shh signaling pathway-related genes

**Fig. 3:**
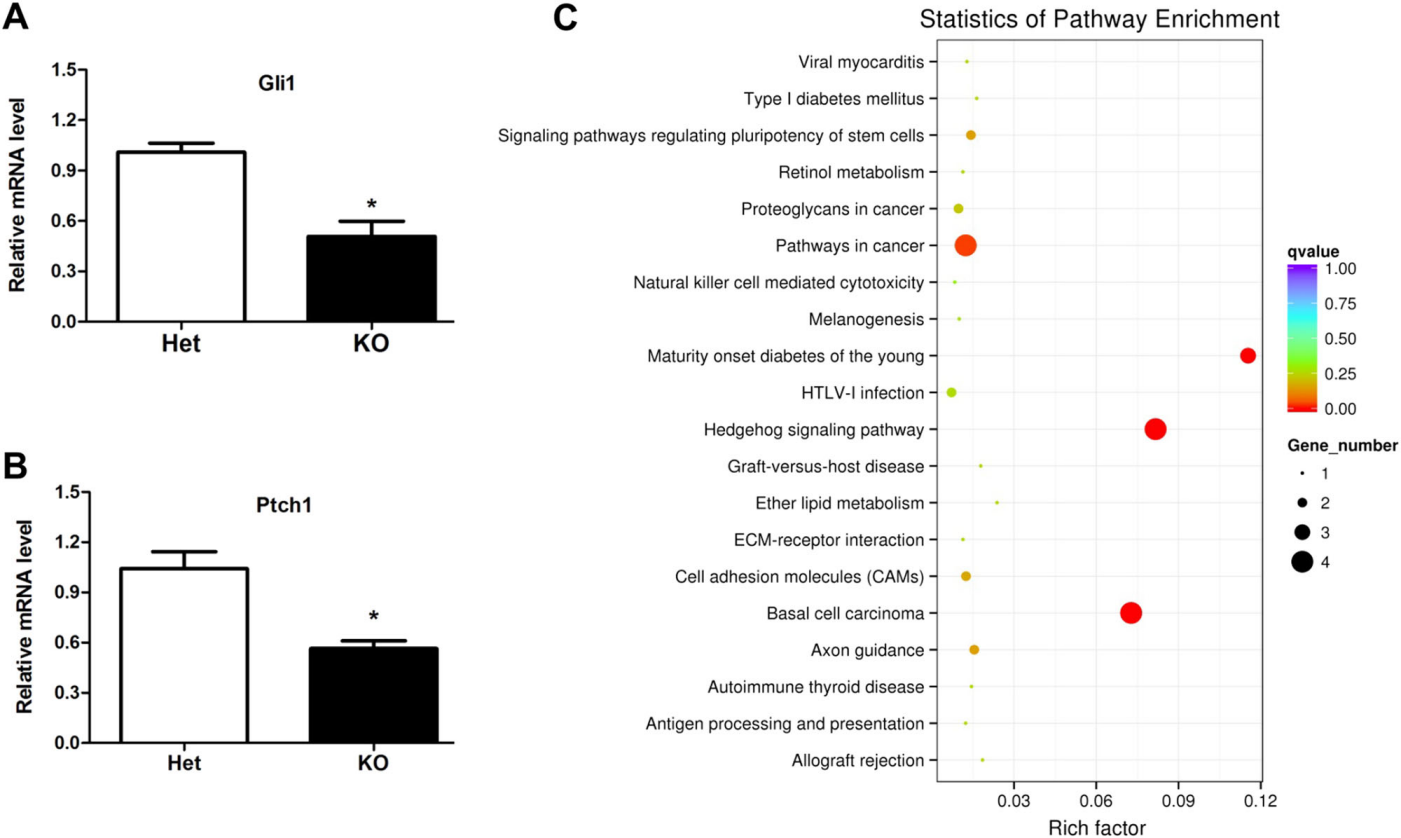
Tctn3 KO results in abnormal expression of the Shh signaling pathway-related genes. At E12.5, total RNA was extracted from mouse brains. The relative mRNA expression of Gli1 (**a**) and Ptch1 (**b**) in Tctn3 Het KO mice and Tctn3 KO mice was examined by quantitative PCR. *P < 0.05 vs Het mice. *N* = 7 (**a**,** b**). At E10.5, embryonic brains were collected for RNA sequencing detection. KEGG enrichment analysis in signaling pathways was performed. *N* = 4 (**c**)

Previous studies have demonstrated that Tctn1, Tctn2, and Tctn3 act as regulators of the Hedgehog signaling pathway^6,8,12^. The Shh signaling pathway is one of the most important pathways in brain development^13,14,37,38^. To examine whether alterations in the Shh signaling pathway were involved in the development of the brain in Tctn3 KO mice, we measured the relative mRNA expression levels of Gli1 and Ptch1, which are directly regulated by the Shh signaling pathway, in Tctn3 KO mice at E12.5. The mRNA expression levels of Gli1 and Ptch1 were significantly reduced in the brains of Tctn3 KO mice compared with Tctn3 Het KO mice (*P* < 0.05, Fig. 3a,b). At the same time, we also collected E10.5 Tctn3 KO and Het KO mice embryos for RNA sequencing detection, showing that 30 genes were obviously down-regulated and four genes were up-regulated in Tctn3 KO mice. Down-regulated genes included Shh and the Shh signaling pathway-related genes, such as Gli1 and Ptch2 (Fig. S4 and Table S1). Kyoto Encyclopedia of Genes and Genomes (KEGG) enrichment analysis indicated that Tctn3 KO affected not only the Shh signaling pathway but also pathways related to carcinogenesis, cell death/survival, and diabetes, as shown in Fig. 3c.

### **Tctn3 KO led to alternation of apoptosis-related protein levels and reduction of neuronal cell numbers in mouse brains**

**Fig. 4:**
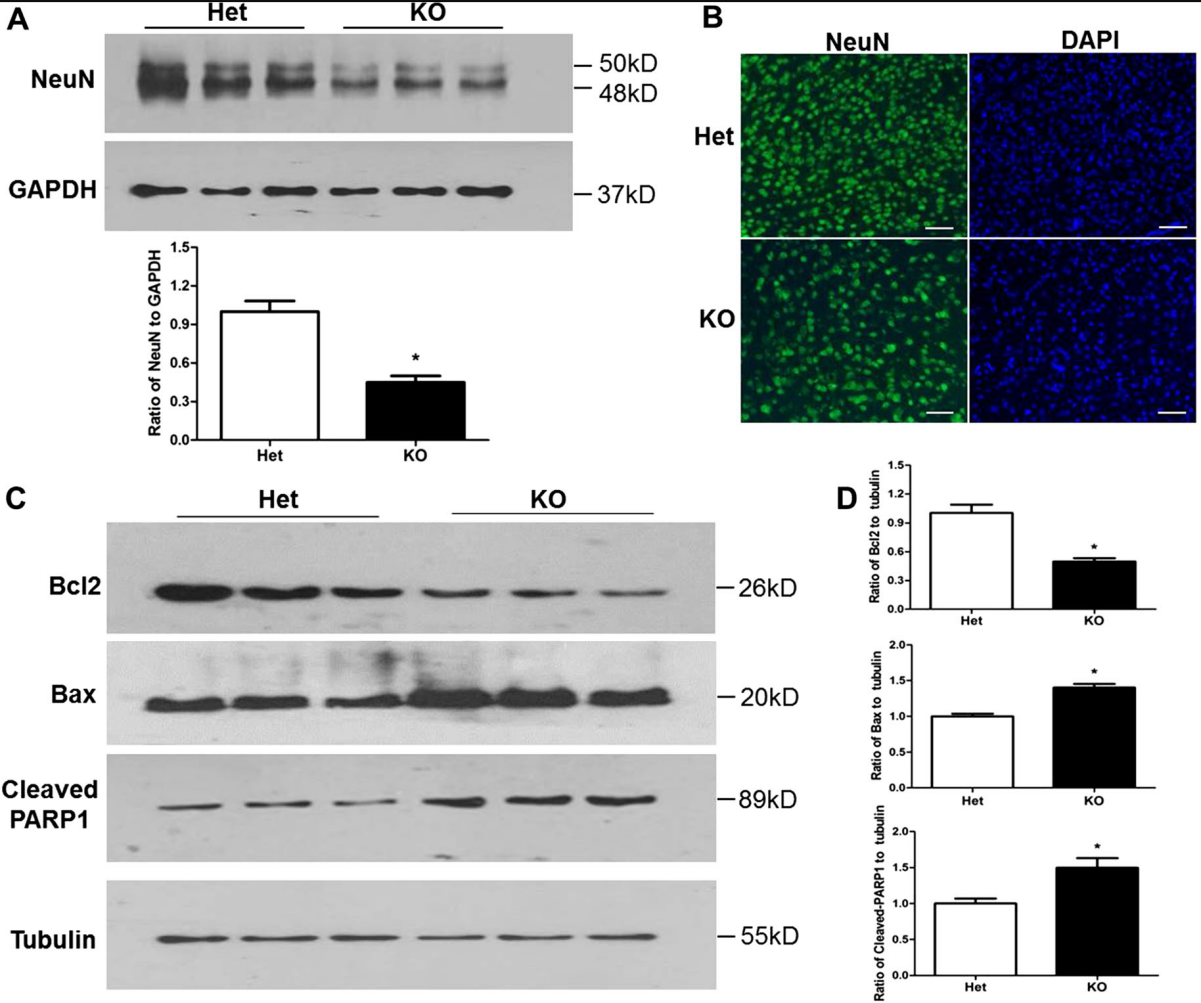
Tctn3 KO led to altered apoptosis-related protein levels in mouse brains. At E12.5, mouse brains were collected, and protein levels of NeuN were determined in Tctn3 Het KO mice and Tctn3 KO mice by Western blot analysis (**a**). At E12.5, mouse brains were fixed, and immunofluorescent staining was performed using anti-NeuN antibody (**b**). The levels of apoptosis-related proteins Bcl2, Bax, and cleaved PARP-1 were examined by Western blot analysis. Beta-tubulin was used as a loading control. *P < 0.05 vs Het mice. *N* = 6. Scale bars: 10 μm

A growing body of evidence suggests that the Shh signaling pathway mediates cell death under physiological and pathological conditions^15,16,18^. To examine whether the Shh signaling pathway affected neuronal cell death during brain development in Tctn3 KO mice, we first examined cell number by levels of the neuronal marker NeuN by immunofluorescent staining and western blotting. Our data indicated that NeuN-positive cells in the brain tissue of Tctn3 KO mice were decreased compared to Tctn3 Het mice (*P* < 0.05, Fig. 4a,b). Further, we detected expression of the apoptosis-related proteins Bcl2, Bax, and cleaved poly (ADP-ribose) polymerase-1 (PARP-1). Our results showed that Bcl2 levels were markedly reduced, but Bax, which is considered one of the determining factors of cell fate, was significantly increased in the brain tissue of Tctn3 KO mice (*P* < 0.05, Fig. 4c,d). In addition, cleaved PARP-1, a substrate of activated caspase-3, was also significantly increased (*P* < 0.05, Fig. 4c,d). These results clearly indicate that Tctn3 KO increases cell death by increasing apoptosis-promoting proteins in the brain.

### Tctn3 KO blocked PI3K/Akt signaling pathway but not the mTOR signaling pathway

**Fig. 5:**
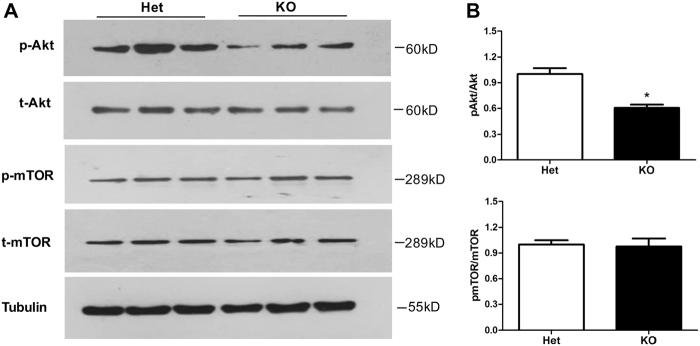
Tctn3 KO inhibited the PI3K/Akt signaling pathway but not the mTOR signaling pathway. At E12.5, mouse brains were collected for Western blot analysis. The expression of p-Akt, total Akt, p-mTOR, and total mTOR in Tctn3 Het KO mice and Tctn3 KO mice were examined (**a**). The relative levels of p-Akt and p-mTOR were quantified. *P < 0.05 vs Het mice. *N* = 6 (**b**)

Shh signals are considered to regulate cell fate through PI3K/Akt and/or mTOR signaling pathways^18,19,39–41^. To examine whether Shh regulates apoptosis by regulating PI3K/Akt and mTOR signals in Tctn3 KO mice, we measured expression of phosphorylated-Akt (p-Akt), total Akt (t-Akt), phosphorylated-mTOR (p-mTOR), and total mTOR (t-mTOR) in the brains of Tctn3 KO mice. Compared with Tctn3 Het KO mice, p-Akt was significantly decreased in the brains of Tctn3 KO mice (*P* < 0.05, Fig. 5a,b), while total Akt did not change (*P* > 0.05, Fig. 5a,b). In contrast, protein levels of p-mTOR and t-mTOR were unchanged in Tctn3 Het KO mice (*P* > 0.05, Fig. 5a,b). These results indicate that Shh regulates apoptosis in the brains of Tctn3 KO mice through a PI3k/Akt-dependent, mTOR-independent pathway.

### The Akt activator SC79 inhibited apoptotic cell death in cultured primary embryonic fibroblasts (pMEFs) from Tctn3 KO mice

**Fig. 6:**
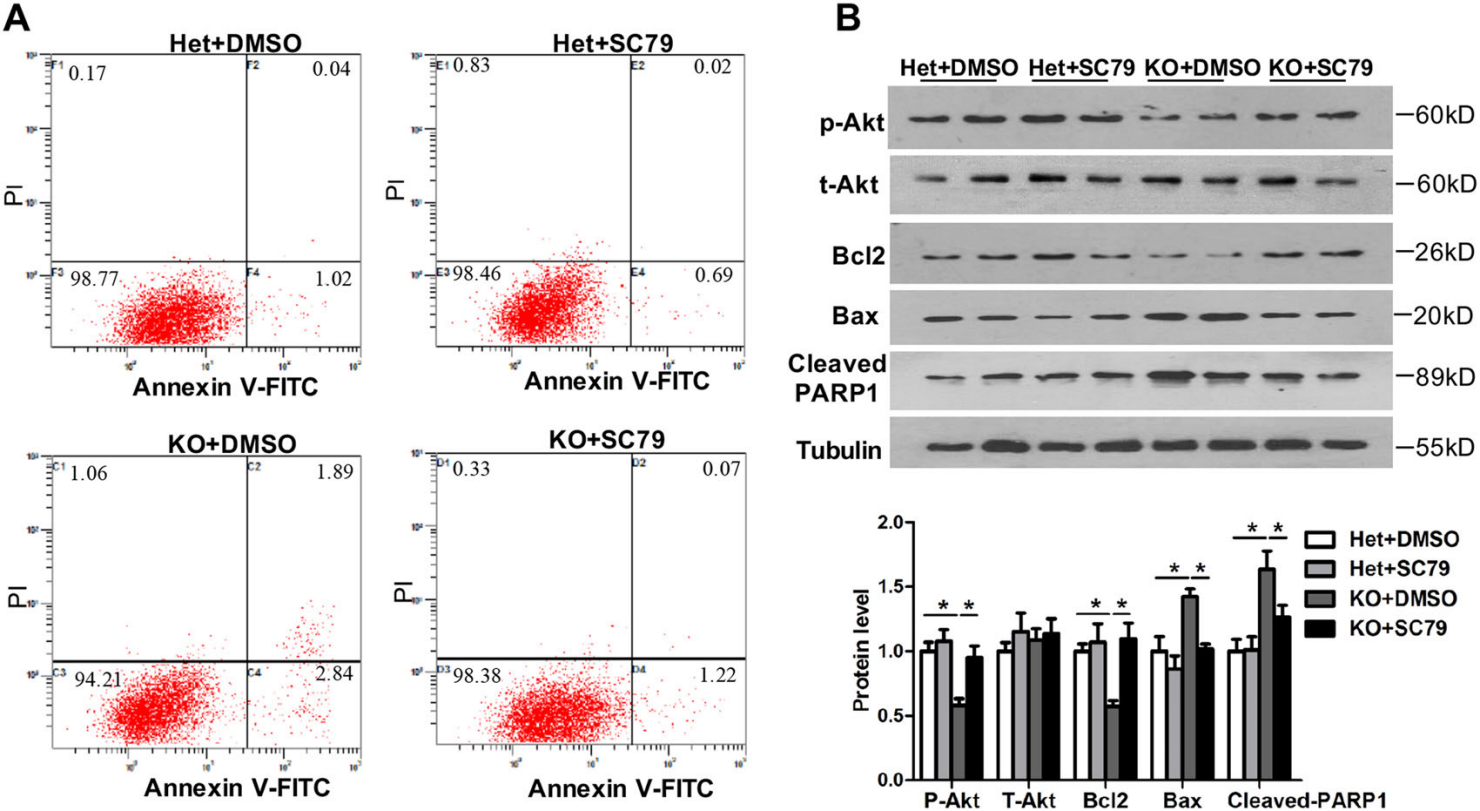
SC79 blocked apoptosis in pMEFs from Tctn3 KO mice. pMEFs from Tctn3 het KO mice and Tctn3 KO mice were cultured at E12.5. pMEFs were incubated with DMSO/SC79 (4 μg/ml) for 24 h. Apoptotic cells were detected by flow cytometry (**a**). At the same time, protein levels of p-Akt, t-Akt, Bcl2, Bax, and cleaved PARP-1 were determined by Western blotting. *P < 0.05 vs Het mice (**b**). *N* = 4

As Tctn proteins are crucial for ciliogenesis in pMEFs^9,10^, we further examined whether knockout of Tctn3 also affects apoptosis of pMEFs. We cultured pMEFs of Tctn3 het and KO mice at E12.5 to explore whether presence of apoptosis in the MEFS of Tctn3 KO mice. Consistent with the presence of elevated apoptosis in the brains of Tctn3 KO mice, Tctn3 KO led to increased levels of apoptotic pMEFs from Tctn3 KO mice as detected by flow cytometry (Fig. 6a). More interestingly, SC79, a small-molecule specific Akt activator^28^, reduced levels of apoptotic pMEFs (*P* < 0.05, Fig. 6a). To further verify this result by Western blot analysis, we found that the expression of p-Akt was also significantly decreased in pMEFs from Tctn3 KO mice (*P* < 0.05, Fig. 6b), while total Akt did not change (*P* > 0.05, Fig. 6b). In contrast, the Akt specific activator SC79 increased p-Akt in pMEFs from Tctn3 KO mice (*P* < 0.05, Fig. 6b). In addition, expression of Bcl2 decreased, while the Bax and cleaved PARP-1 increased in pMEFs from Tctn3 KO mice (*P* < 0.05, Fig. 6b), and these changes were reversed by treatment with SC79 (*P* < 0.05, Fig. 6b). These results indicated that apoptosis in pMEFs from Tctn3 KO mice is mediated, at least partially, by a PI3k/Akt-dependent pathway.

### Tctn3 KO decreased NPHP1 expression in mouse brains

**Fig. 7:**
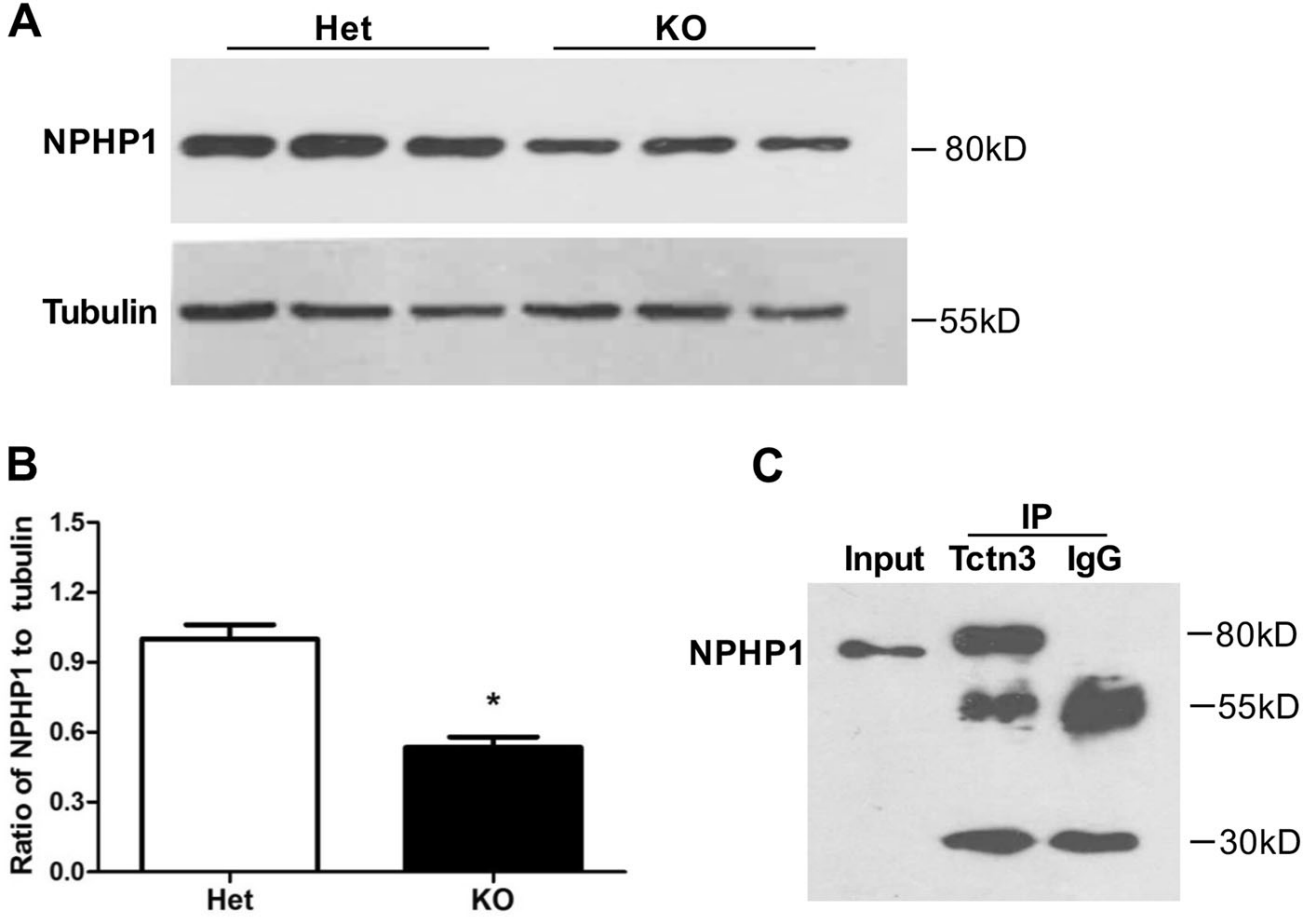
Tctn3 KO decreased NPHP1 expression at E12.5. Mouse brains were collected at E12.5 for the detecting of NPHP1 expression by western blot analysis. Representative picture was shown (**a**). Quantitative analysis of NPHP1 expression was performed. *P < 0.05 vs Het mice. *N* = 6 (**b**). To demonstrate the interaction of Tctn3 and NPHP1, immunoprecipitation was carried out using an anti-Tctn3 antibody (**c**)

NPHP1 is a cytoplasmic adaptor molecule that localizes to cilia, cell-cell junctions, and cell-matrix adhesion sites^20,22,42^. NPHP1 deficiency or mutation also causes ciliopathies such as JBTS, NPHP or BBS^43–48^. Previous studies have demonstrated that both Tctn3 and NPHP1 interact with Tctn1 and/or Tctn2^4,10,12^. Intriguingly, NPHP1 also regulates resistance to apoptosis but not the cell cycle^22^. To ascertain whether NPHP1 expression is altered in the brains of Tctn3 KO mice, we collected brain tissues from E12.5 mice to examine the protein expression of NPHP1 in Tctn3 KO mice. Our results showed that NPHP1 expression in the brains of Tctn3 KO mice was significantly decreased compared with that in Tctn3 Het KO mice (*P* < 0.05, Fig. 7a,b). To further investigate whether Tctn3 potentially interacts with NPHP1 to form a complex that plays a critical role in apoptosis, we performed immunoprecipitation of NPHP1 and Tctn3 from brain tissues. Our results indicated that NPHP1 interacts with Tctn3 (Fig. 7c). Therefore, Tctn3 and NPHP1 may function together to regulate cell death and survival, but this hypothesis needs more investigation.

## Discussion

### **Tectonic proteins play vital roles in neural tube development by mediating the Shh signaling pathway in mice**

The tectonic protein family is a group of TZ proteins, including Tctn1, Tctn2, and Tctn3, which comprise one membrane binding protein and two single-pass membrane proteins^8,9^. The Shh signaling pathway is one of the most important developmental mediators^13,14,37,38^. Although tectonic proteins regulate the Hedgehog signaling pathway^6,8,12^ and its downstream signals such as Gli1 and Ptch1, the three tectonic proteins still display different roles in mediating the Shh pathway and development in mice. Tctn1 null mice present with heterotaxia, holoprosencephaly, microphthalmia, and hind limb polydactyly, similar to the phenotypes of mice with Shh signaling defects^4,8^. Apart from these characteristics of Tctn1 mice, Tctn2^−/−^ mice present additional phenotypes, including cleft palate, right-sided stomach, and ventricular septal defect^12^. In our study, Tctn3 mutant embryos display edema on the back of the upper body and randomized heart looping^9^, as well as polydactyly (Fig. 1c,d), abnormal eyes (Fig. 1b) and abnormal skull and sternum (Fig. S1A, B). The polydactyly phenotype that has been reported is closely associated with changes in the Shh signaling pathway^49^. Based on the phenotype of mutant mice, we speculate that all three members of the Tctn family have various functions in development and regulation of the Shh signaling pathway.

In addition, several molecular markers regulated by the Shh signal were changed in Tectonic mutant mouse embryos^8,9,12^. Tctn1^−/−^ and Tctn2^−/−^ mice present with significantly decreased Islet1/2-positive motor neurons, while Pax6, a molecule suppressed by Shh, is widely distributed^8,12^. In this study, although Tctn3 KO mice exhibit normal Pax3 patterning in the dorsal neural tube, Shh, Foxa2, Nkx2.2, Olig2, Hb9, Islet1/2, and Pax6 patterning are significantly ventrally shifted. In addition, the Arl13b protein, a marker of cellular cilia, was significantly decreased in Tctn3 KO mice (Fig. 2). In a recent report, Wang et al.^9^ found that the Shh signal pathway was disrupted in Tctn3 null mice, as shown by undetectable Foxa2 and ventrally shifted Nkx2.2; however, Pax6, Pax7, and Hb9 distributions were normal, which was different from our results. Therefore, our Tctn3 KO mice seemed to have more severe symptoms. For example, these mice had prenatal lethality, microphthalmia, and bone changes. This inconsistency may be explained by different approaches to create the Tctn3 KO. In the previous report, mutant Tctn3 mice were produced by deleting the exon 3 of Tctn3 with a Cre-Loxp system. In the present study, Tctn3 mutant mice were generated by insertional mutagenesis. In summary, we conclude that Tectonic proteins play vital roles in neural tube development and the Shh signaling pathway.

### Tctn3 regulates apoptosis during brain development in mice

Although the three Tctn proteins have high levels of homology^9^, the role of Tctn3 in the Shh signaling pathway and development is distinct from that of Tctn1 and Tctn2. This raises an interesting question as to whether Tctn3 KO mice have other phenotypes. Increasing evidence indicates that the Shh signaling pathway plays a significant role in inhibiting apoptosis^15,16,18^. The decline in the Shh signaling pathway (Fig. 3a,b) in the brains of Tctn3 KO mice was associated with obvious reduction in NeuN-positive cells (Fig. 4a,b) and the anti-apoptotic protein Bcl2, as well as increases in the apoptotic markers Bax and cleaved PARP-1 (Fig. 4c). These results clearly indicate that Tctn3 KO leads to increased apoptosis in mouse brains.

Many studies have shown that the Shh signaling pathway is involved in the regulation of cell fate through PI3K/Akt and/or mTOR signaling pathways^18,19,39–41^. In this study, p-Akt was significantly decreased in the brains of Tctn3 KO mice, but p-mTOR was not changed (Fig. 5a, b). In addition, both Tctn3 and NPHP1 have been shown to interact with Tctn1 and/or Tctn2^4,10,12^. Interestingly, NPHP1 can also regulate resistance to apoptosis but not the cell cycle in mammals^22^. Protein expression of NPHP1 was also significantly decreased in the brains of Tctn3 KO mice (Fig. 7a,b), and NPHP1 physically interacts with Tctn3 (Fig. 7c). These results suggest that NPHP1 may also be involved in apoptosis induced by Tctn3 KO.

To explore whether increased apoptosis in Tctn3 KO mice could be prevented by Akt agonists, we cultured pMEFs, in which ciliogenesis is regulated by Tctn3 at E12.5^9^. The pMEFs from Tctn3 KO mice exhibited an increase in apoptosis, as detected by flow cytometry analysis (Fig. 6a). Use of SC79, a small-molecule unique specific Akt activator^28^, elevated levels of p-Akt in pMEFs from Tctn3 KO mice (Fig. 6b) and reduced apoptosis in pMEFs from Tctn3 KO mice (Fig. 6). These results indicated that apoptosis in pMEFs from Tctn3 KO mice is mediated by a PI3k/Akt-dependent pathway.

### Tectonic mutations-related diseases

Destruction of the integrity of ciliated protein complexes leads to defects in the cilium membrane protein components, leading to many human ciliopathies^1–3^. As one of the important cilial network proteins, Tectonic mutations are also known to cause different ciliopathies, including MKS, OFDS, and JBTS^50–52^. These ciliopathies are developmental diseases that follow a recessive pattern, with no symptoms found in carriers of the mutation, which is consistent our Tctn3 het KO mouse data (Figs. S2–3). As structural proteins, Tectonic proteins are considered to traffic proteins into the cilia so that they play an essential role in regulating ciliary membrane composition and ciliogenesis^10^. At the same time, Tectonic proteins are connected with components of some important signal pathways, such as the Shh signaling pathway and apoptosis-related signaling pathways, as evidenced in this study. Different mutations in Tectonic genes may cause loss or gain of functions resulting in different phenotypes and heterogeneity in ciliopathies^53^. However, the underlying mechanisms of connections between Tectonic proteins and different important signaling pathways remain unclear. Understanding the molecular pathogenesis of Tectonic-induced ciliopathies may help treat patients with ciliopathies and improve their quality of life.

## Conclusion

In summary, Tctn3 deficiency disrupts the Shh signaling pathway and the neural tube patterning, resulting in abnormal embryonic development and prenatal death in mice. Therefore, Tctn3 has distinct roles in the normal development of mouse embryos including brains, eyes, and bones that could not be compensated by Tctn1 and Tctn2 proteins.

## Electronic supplementary material

Table S1

Figure S1

Figure S2

Figure S3

Figure S4

Supplementary figure legends
