# Supplementary material for: Loss of Tctn3 causes neuronal apoptosis and neural tube defects in mice
Source: Cell Death Dis. 2018 May 3;9(5):520. doi: 10.1038/s41419-018-0563-4 (PMC5938703; doi:10.1038/s41419-018-0563-4)
Supplement: Supplementary file 1 — Table S1 [file 41419_2018_563_MOESM1_ESM.docx]

**Table S1 RNA sequencing results in Tctn3 KO and Het KO mouse embryos at E10.5.**

**Down regulated genes**

| Gene ID | Gene Name | Description | P value |
| --- | --- | --- | --- |
| ENSMUSG00000001496 | **Nkx2-1** | **NK2 homeobox 1** | **8.50E-09** |
| ENSMUSG00000002633 | **Shh** | **sonic hedgehog** | **1.84E-07** |
| ENSMUSG00000012520 | **Phox2b** | **paired-like homeobox 2b** | **7.61E-07** |
| ENSMUSG00000016918 | **Sulf1** | **sulfatase 1** | **1.07E-07** |
| ENSMUSG00000017344 | **Vtn** | **vitronectin** | **3.22E-05** |
| ENSMUSG00000020598 | **Nrcam** | **neuronal cell adhesion molecule netrin 1** | **5.48E-05** |
| ENSMUSG00000020902 | **Ntn1** | **ectonucleotide** | **7.53E-06** |
| ENSMUSG00000022425 | **Enpp2** | **pyrophosphatase/phosphodiesterase2** | **1.28E-05** |
| ENSMUSG00000025020 | **Slit1** | **slit homolog 1 (Drosophila)** | **5.69E-28** |
| ENSMUSG00000025407 | **Gli1** | **GLI-Kruppel family member GLI1** | **2.14E-05** |
| ENSMUSG00000027434 | **Nkx2-2** | **NK2 homeobox 2** | **5.83E-09** |
| ENSMUSG00000028681 | **Ptch2** | **patched homolog 2** | **1.31E-05** |
| ENSMUSG00000033726 | **Emx1** | **empty spiracles homeobox 1** | **4.74E-05** |
| ENSMUSG00000034648 | **Lrrn1** | **leucine rich repeat protein 1, neuronal** | **6.34E-05** |
| ENSMUSG00000036961 | **Wnt8b** | **wingless-type MMTV integration site family, member 8B** | **2.78E-08** |
| ENSMUSG00000037025 | **Foxa2** | **forkhead box A2** | **2.40E-16** |
| ENSMUSG00000038156 | **Spon1** | **spondin 1, (f-spondin) extracellular matrix protein** | **5.14E-16** |
| ENSMUSG00000041309  ENSMUSG00000046518 | **Nkx6-2**  **Ferd3l** | **NK6 homeobox 2**  **Fer3-like (Drosophila)** | **3.37E-09**  **4.33E-07** |
| ENSMUSG00000047261 | **Gap43** | **growth associated protein 43** | **5.46E-05** |
| ENSMUSG00000049538 | **Adamts16** | **a disintegrin-like and metallopeptidase (reprolysin type) with thrombospondin type 1 motif, 16** | **6.53E-06** |
| ENSMUSG00000053279 | **Aldh1a1** | **aldehyde dehydrogenase family 1, subfamily A1** | **9.20E-14** |
| ENSMUSG00000054160 | **Nkx2-4** | **NK2 homeobox 4** | **2.97E-06** |
| ENSMUSG00000058669 | **Nkx2-9** | **NK2 homeobox 9** | **9.91E-15** |
| ENSMUSG00000068428 | **Gmnc** | **geminin coiled-coil domain containing** | **5.56E-05** |
| ENSMUSG00000074607 | **Tox2** | **TOX high mobility group box family member 2** | **1.22E-05** |
| ENSMUSG00000097156 | **Gm3764** | **predicted gene 3764** | **2.28E-05** |
| Novel00066 | **--** |  | **2.70E-09** |
| Novel00073 | **-//-** |  | **1.97E-05** |
| Novel00207 | **--** |  | **8.74E-11** |

**Up regulated genes**

| Gene ID | Gene Name | Description | P value |
| --- | --- | --- | --- |
| ENSMUSG00000027168 | **Pax6** | **paired box 6** | **1.63E-05** |
| ENSMUSG00000029697 | **Fezf1** | **Fez family zinc finger 1** | **8.16E-06** |
| ENSMUSG00000037161 | **Mgarp** | **mitochondria localized glutamic acid rich protein** | **1.60E-05** |
| ENSMUSG00000061232 | **H2-K1** | **histocompatibility 2, K1, K region** | **3.63E-05** |
