# Supplementary figures and images for: Loss of Tctn3 causes neuronal apoptosis and neural tube defects in mice
Source: Cell Death Dis. 2018 May 3;9(5):520. doi: 10.1038/s41419-018-0563-4 (PMC5938703; doi:10.1038/s41419-018-0563-4)

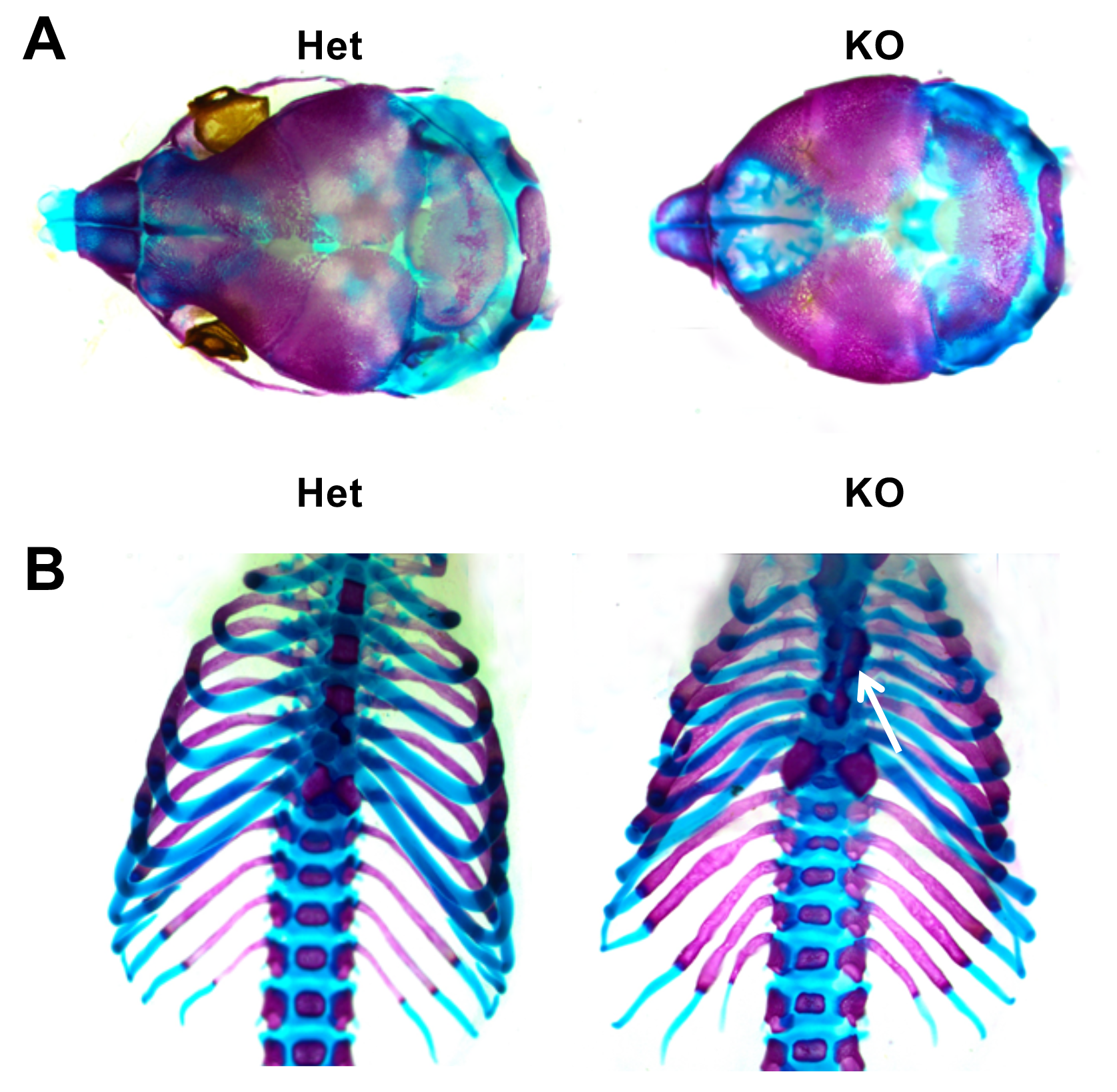

Supplement: Supplementary file 2 — Figure S1 [file 41419_2018_563_MOESM2_ESM.tif]

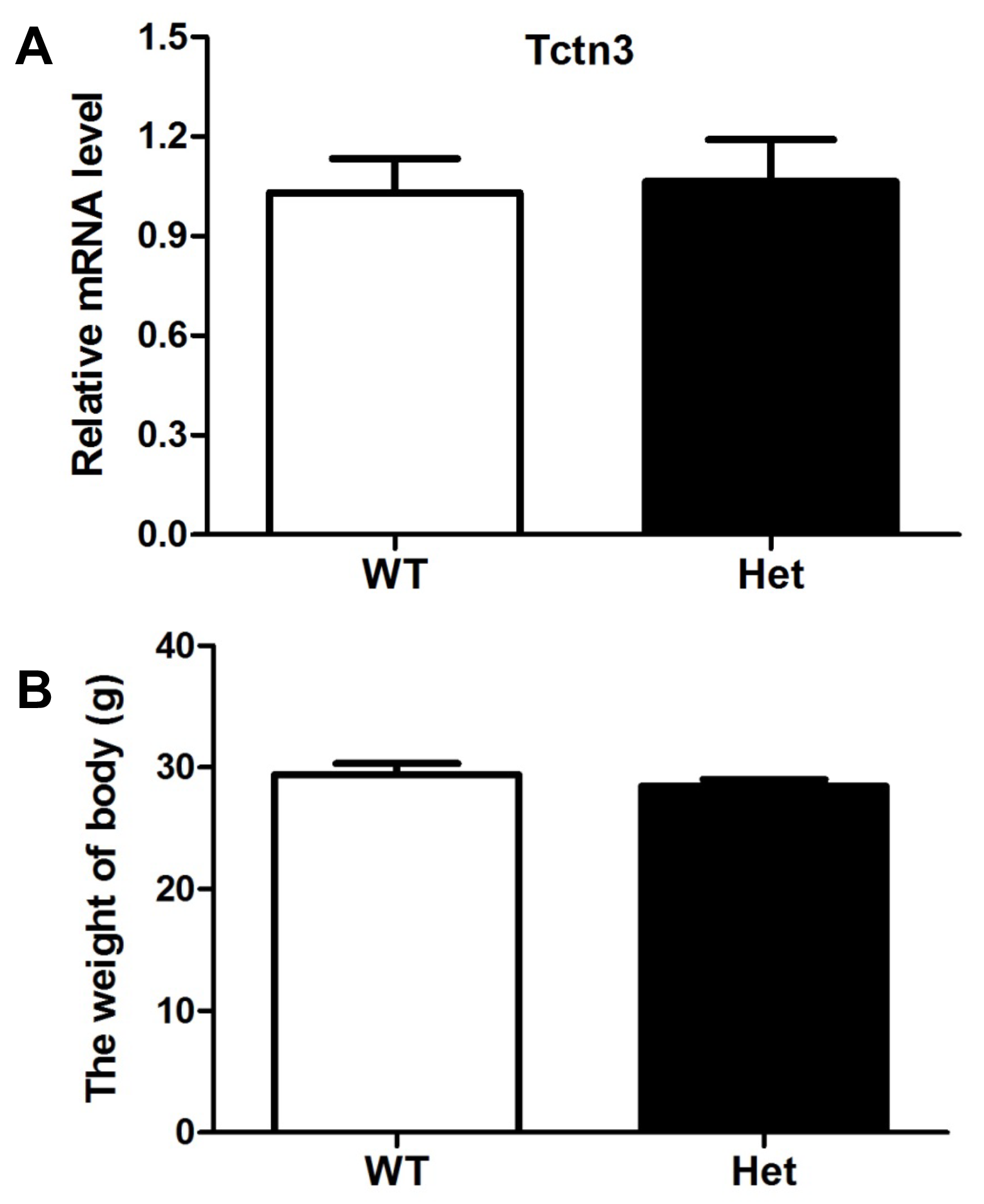

Supplement: Supplementary file 3 — Figure S2 [file 41419_2018_563_MOESM3_ESM.tif]

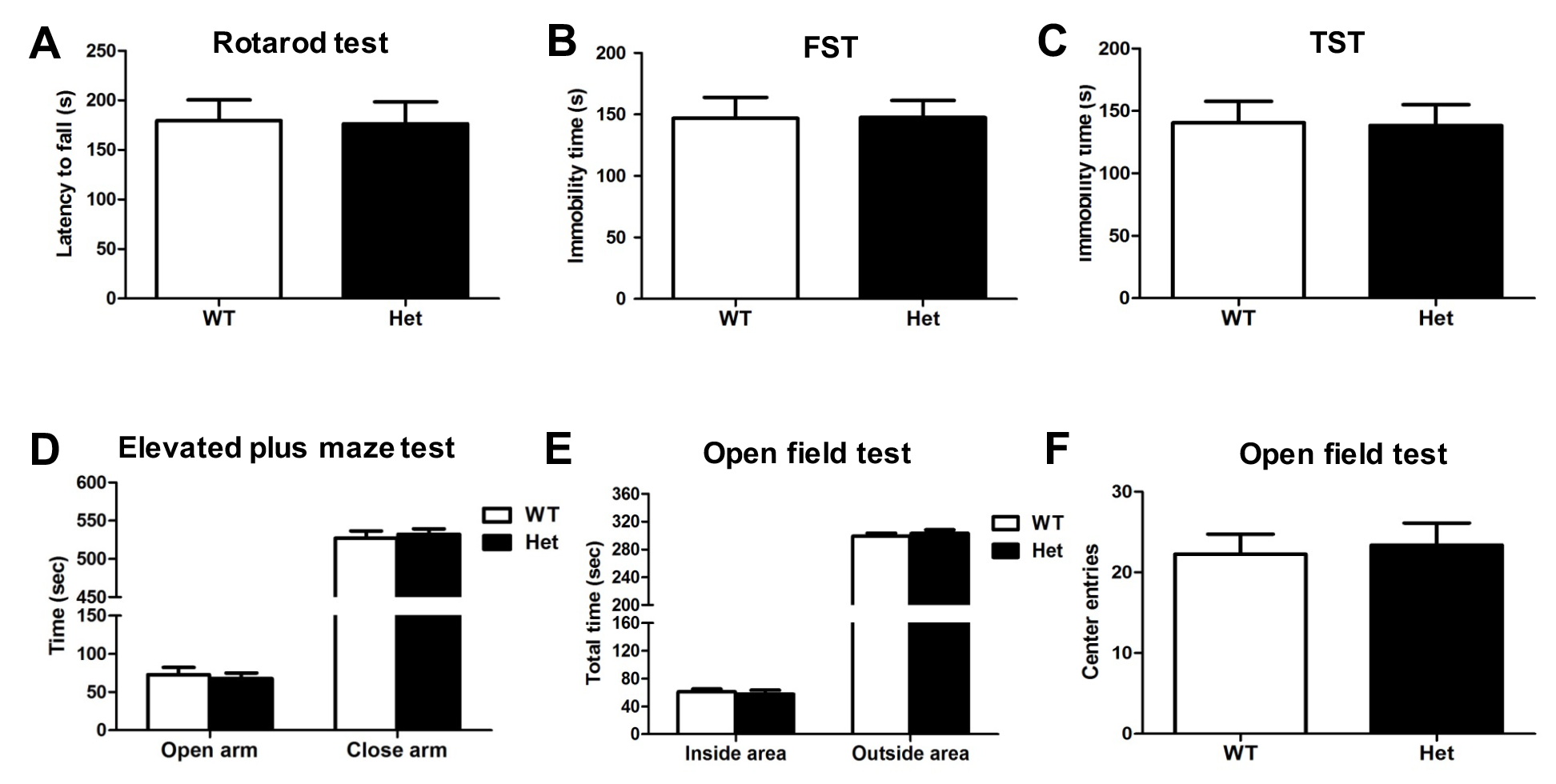

Supplement: Supplementary file 4 — Figure S3 [file 41419_2018_563_MOESM4_ESM.tif]

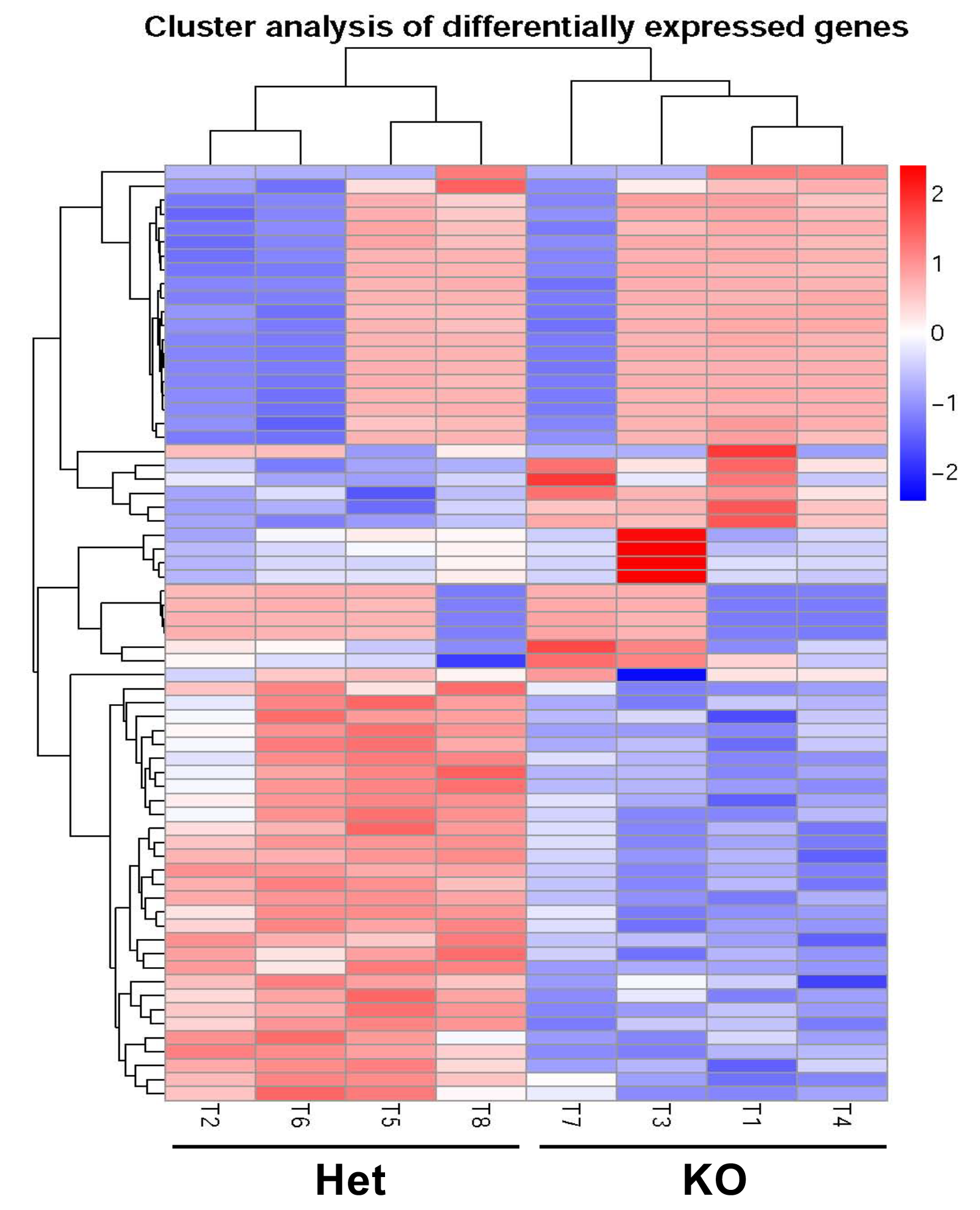

Supplement: Supplementary file 5 — Figure S4 [file 41419_2018_563_MOESM5_ESM.tif]
