# Supplementary material for: Loss of Tctn3 causes neuronal apoptosis and neural tube defects in mice
Source: Cell Death Dis. 2018 May 3;9(5):520. doi: 10.1038/s41419-018-0563-4 (PMC5938703; doi:10.1038/s41419-018-0563-4)
Supplement: Supplementary file 6 — Supplementary figure legends [file 41419_2018_563_MOESM6_ESM.docx]

**Loss of Tctn3 causes neuronal apoptosis and neural tube defects in mice**

Bin Wang^1,2#^, Yingying Zhang^1#^, Hongli Dong^3#^, Siyi Gong^1,2^, Bin Wei^1^, Man Luo^2^, Hongyan Wang^4,5^, Xiaohui Wu^5,6^, Wei Liu^7^, Xingshun Xu^2*^, Yufang Zheng^4,5,6*^, Miao Sun^1*^

^1^Institute for Fetology, the First Affiliated Hospital of Soochow University, Suzhou City 215006, Jiangsu, China; ^2^Institute of Neuroscience, Soochow University, Suzhou City 215123, Jiangsu, China; ^3^Department of Neurology, Suzhou Hospital of Traditional Chinese Medicine; ^4^Obstetrics and Gynecology Hospital Research Center, Institute of Reproduction and Development, Fudan University, Shanghai 200433, China; ^5^State Key Laboratory of Genetic Engineering, MOE Key Laboratory of Contemporary Anthropology, and Collaborative Innovation Center for Genetics & Development, School of Life Sciences, Fudan University, Shanghai 200438, China; ^6^Institute of Developmental Biology & Molecular Medicine, Fudan University, Shanghai 200433, China; ^7^Department of Pathology, the First Affiliated Hospital of Soochow University, Suzhou City 215006, Jiangsu, China

# BW, YZ, and DH contributed equally to this work.

*Correspondence should be addressed to Prof. Xingshun Xu (xingshunxu@suda.edu.cn), Prof. Yufang Zheng (zhengyf@fudan.edu.cn), or Prof. Miao Sun (miaosunsuda@163.com)

**Supplemental figure legends**

**Fig. S1 Tctn3 KO led to abnormal development of the skull and sternum in mice.** At postnatal stage P0, mice were collected for bone staining. Alizarin red S and alcian blue staining show dome-shaped heads with a hole (A) and abnormal bony fusion of sternum (white arrow, B) in Tctn3 KO mice.

**Fig. S2 Expression of Tctn3 mRNA and body weight did not change in Tctn3 Het KO mice.** At E12.5, mouse embryos were collected, and relative mRNA of Tctn3 in the embryos of Tctn3 Het KO mice and WT mice were examined by quantitative PCR. N=6 (A). At 2 m, body weight was measured in Tctn3 Het KO mice and wild type (WT) mice by an electronic balance. N=8 (B).

**Fig. S3 Tctn3 Het KO mice did not exhibit movement deficiency, depression-like or anxiety-like behaviors.** After mice were 2 months old, a series of behavioral tests were performed in Tctn3 Het KO mice and WT mice. Latency to fall time was recorded in the rotarod test (A). Immobility time in the tail suspension test (B) and forced swimming test (C) was recorded. In the elevated plus maze test, the time mice spent in open arms and closed arms was recorded (D). In the open field test, the total time spent in the inside area and outside area was recorded (E) and center entries was also recorded (F). N=8.

**Fig. S4 Tctn3 KO caused changes of genes** **expression.** At E10.5, Tctn3 KO and Het mice embryos were collected for RNA sequencing detection. Cluster analysis of differential gene expression was performed. N=4.
